# Is diabetes the risk factor for poor neurological recovery after cervical spine surgery? A review of the literature

**DOI:** 10.1186/s40001-022-00879-6

**Published:** 2022-11-23

**Authors:** Peng Wang, Baoge Liu, Tianhua Rong, Bingxuan Wu

**Affiliations:** 1 Department of Orthopaedic Surgery, Beijing Tiantan Hospital, Capital Medical University, No. 119 South 4th Ring West Road, Fengtai District, Beijing, 100070 China; 2 Department of Orthopedics, Jinan Central Hospital, Shandong First Medicine University, 250013 Jinan, China

**Keywords:** Diabetes, Microangiopathy, Microvasculitis, Hyperostotic spondylosis, Diabetic neuropathy, Diabetic polyneuropathy, Diabetic radiculoplexus neuropathy

## Abstract

The poor prognosis of cervical spine surgery is mainly manifested as poor neurological recovery and the presence of new upper extremity dysfunction that promotes significant psychological and physiological burdens on patients. Many factors influence the prognosis of cervical spine surgery, including the age of patients, the time and mode of surgery, and the surgical technique used. However, in clinical studies, it has been observed that patients with diabetes have a higher probability of poor prognosis after surgery. Therefore, we review the pathophysiology of diabetic neuropathies and discuss its impact on cervical nerve system function, especially in cervical nerve roots and upper limb peripheral nerve conduction.

## Background

Some cervical spondylosis patients exhibit unsatisfactory outcomes after surgery and experience poor neurological recovery, including the development of new nerve palsy that did not exist before surgery; these outcomes are especially observed in those with comorbid diabetes. The main factors affecting the efficacy of cervical spine surgery include hypertension, advanced age, decompression and fusion of multiple segments, overextension of the intraoperative intervertebral space, postoperative spinal cord drift, stretching of the nerve root, and ischaemic reperfusion injury [1–6]. However, the impact of diabetes on surgical outcome, especially during neurological recovery, is still not well elucidated.

Diabetic neuropathy is one of the most common complications of diabetes [7, 8]. It is mainly divided into diabetic distal symmetric polyneuropathy and asymmetric neuropathies [9–14], including more than ten subtypes according to clinical symptoms. Diabetes affects not only the peripheral nervous system, but also the central nervous system [15]. A long-term diabetes course and/or poor glucose control has been shown to lead to structural changes and functional impairment of the spinal cord and nerve roots, and glucose correction during the perioperative period cannot completely prevent poor neurological recovery after surgery.

Therefore, it is very necessary to better understand the impact of abnormal glucose metabolism on cervical spondylosis patients with diabetes, which can be applied correctly to judge surgical indications to evaluate the prognosis.

## Pathophysiology of diabetic neuropathy

### Diabetic polyneuropathy (DPN)

Diabetic polyneuropathy (DPN) is a type of diabetic distal symmetric polyneuropathy, which is a multiple neuropathy characterised by the abnormal sensation and movement of both lower limbs. It is predominantly a sensory impairment, but motor impairment may also occur in severe stages of the disease. In patients with type 1 diabetes, DPN usually occurs several years or more after the onset of hyperglycaemia. However, at the time of the formal diagnosis of type 2 diabetes, DPN may already be present. Patients with DPN can develop length-dependent sensorimotor polyneuropathy, the pathogenic characteristics of which include persistent hyperglycaemia, microvascular dysfunction, oxidative and nitrosative stress, neurotrophic deficiencies, and autoimmune-mediated neurological destruction [16, 17]. Demyelination, axonal loss, and gliosis can occur in cranial, spinal, and distal nerves. Large nerve fibres in peripheral nerves mediate movement, sensation, vibration, and proprioception, whereas smaller fibres mediate pain and temperature sensation. Peripheral neuropathy involves sensory, motor, and autonomic nerve fibres, and the disease can affect nerves unilaterally or bilaterally, resulting in sensory abnormalities (loss, hypersensitivity, pathological pain) and, in a few patients, decreased muscle strength. Because the onset of DPN is often insidious, slow, and unnoticeable, it is often ignored when symptoms are early or mild.

#### Demyelination and axonal degeneration

**Fig. 1:**
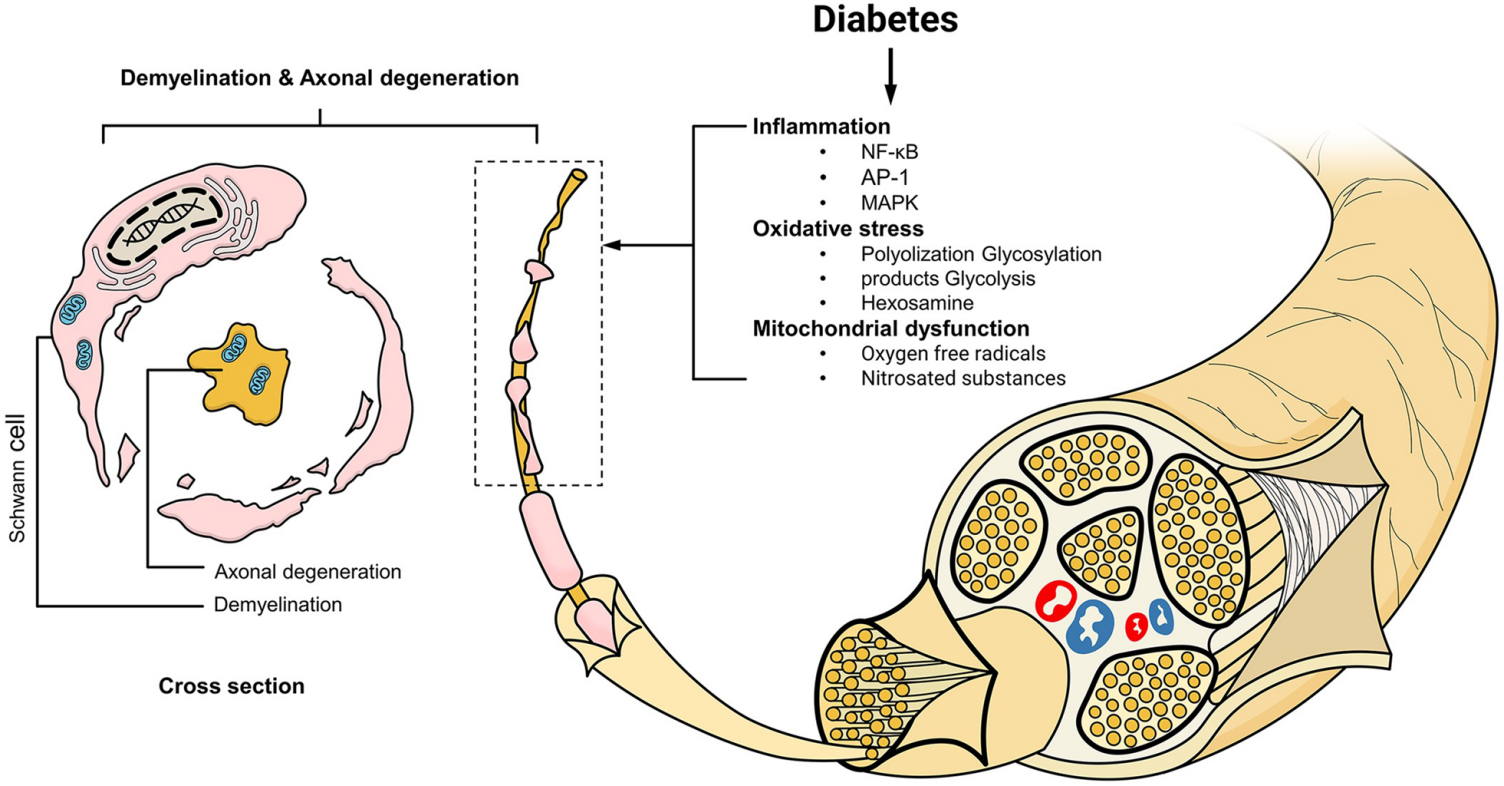
Pathological schematic diagram of diabetic peripheral neuropathy

The pathologies exhibited in patients with DPN include inflammation, oxidative stress response, and mitochondrial dysfunction. Inflammation induces increases in the concentrations of nuclear factor kappa B (NF-κB), activator protein 1 (AP-1), and mitogen-activated protein kinase (MAPK). Oxidative stress is mediated through multiple pathways, including those involving glycosylation products, glycolysis, and hexosamine. Mitochondrial dysfunction is responsible for the production of reactive oxygen species and nitrosated substances. Free radicals cause lipid peroxidation, protein modification, and nucleic acid damage, ultimately leading to axonal degeneration and changes in segmental demyelination (Fig. 1) [18–22]. Axonal degeneration is the main problem occurring in DPN, resulting in altered neurological function due to chronic hyperglycaemia. DPN can be partially prevented by promoting normoglycemia [23].

#### Microangiopathy

Persistent hyperglycaemia in patients with diabetes produces reactive oxygen species and reactive nitrogen species, which are recognised causes of the macrovascular and microvascular complications of diabetes [24, 25]. The blood vessels of diabetic patients undergo a process of repeated injury, regeneration and healing, and these reactive oxygen species and reactive nitrogen species can spread throughout the blood vessels of the body and cause serious cardiovascular disease. This process eventually leads to peripheral vascular disease, affecting the normal function of both large blood vessels and microvessels throughout the body [26, 27]. Microvascular disease is common in patients with diabetes. Fagerberg [28] found as early as 1957 that the axons of peripheral nerves depend on the blood supply from the microenvironment and that chronic hyperglycaemia causes the thickening of the capillary basement membrane and occlusive vasculopathy, hypoxia, and damage to nerve tissue, which are structural features of diabetic microangiopathy. Wietecha et al. [29] found that diabetic patients exhibit thickened microvascular walls with impaired saturation and that the functions of multiple vascular repair factors are inhibited, suggesting that diabetes delays the maturation of reconstructed microvasculature and damages its integrity and stability. Research has also shown that even in the early stages of the disease, dysfunction of the large blood vessels and microvasculature reduces the oxygen supply per unit of time. This finding explains the hypoxic damage to nerve tissue that is observed despite normal or elevated blood flow in peripheral nerves. Nerve biopsy also reveals microvascular defects, including the thickening of the nerve basement membrane and endothelial cell proliferation and hypertrophy, and more severe pathological changes occur in the capillaries within the nerve [30–38]. In contrast, axonal degeneration, secondary loss of Schwann cells and misdirection of regenerating fibres are the causes of defective neural regeneration [39].

### Diabetic radiculoplexus neuropathy (DRPN)

Diabetic radiculoplexus neuropathy (DRPN) is a neurological complication of diabetes mellitus, characterised by the sudden onset of pain and subsequent muscle weakness and sensory abnormalities, and it may be accompanied by autonomic nervous dysfunction. The disease is often unilateral and focal at first but may progress to bilateral, multifocal lesions or even to distant innervated areas. The most common form of DRPN is diabetic lumbosacral radiculoplexus neuropathy (DLRPN), and as the disease progresses, the thoracic and cervical nerve roots are also involved. Dyck and Windebank [40] showed that 10% of patients with DLRPN also had diabetic cervical radiculoplexus neuropathy (DCRPN) or diabetic thoracic radiculoneuropathy (DTRN). Dyck et al. [2] found that 1/3 of patients with DLRPN exhibited nerve involvement of the upper limb, with some patients showing multinerve involvement, consistent with cervical radiculopathy (DCRPN). These mainly included the median and ulnar nerves. In the course of the disease, motor, sensory and autonomic nerves may all be involved. Patients with DLRPN also experience pain in the upper limbs and chest. DCRPN and DTRN may share the same pathophysiological basis as DLRPN (ischaemic injury and microvasculitis), as they often occur together in the same patient. DCRPN and DTRN can also be exhibited alone without DLRPN. Many of the unilateral neuropathies of the upper limbs in DLRPN may be associated with weight loss and compressive injuries secondary to wheelchair dependence. In DRPN patients, cerebrospinal fluid (CSF) protein concentrations are usually elevated, indicating that the disease process extends to the nerve root level [2]. Patients with DRPN may exhibit an increased erythrocyte sedimentation rate and a rheumatoid factor response and positivity for antinuclear antibodies or other markers of immune-mediated disease. Electroneurophysiological examination of the DLRPN revealed reduced wave amplitude and prolonged latency with involvement of the lower limbs and paravertebral muscles. Patients with simple electrophysiological abnormalities are more common than those with obvious clinical manifestations. Quantitative sensory and autonomic tests show that fibres of all sizes are involved and that the disease affects the sensory, autonomic and motor nervous systems. The histopathological abnormalities observed in DLRPN are consistent with the primary axonal degeneration caused by ischaemic nerve injury. The pathology of the distal cutaneous nerve exhibits multifocal fibre loss, perineural degeneration and thickening, failure of neovascularisation and nerve fibre regeneration, and formation of microfascia. These findings are consistent with ischaemia. The increased incidence of axonal degeneration and presence of empty nerve endings occurs, with segmental demyelination often clustered in individual nerve fibres (with evidence that the extent of demyelination is second only to the level of axonal dystrophy) [2, 40]. Unlike in DPN, peripheral cell degeneration and basement membrane overlap are usually not visible in the microvasculature of DLRPN neural tissue [40]. In one study, 142 DLRPN patients exhibited a shorter time of diabetes before onset than that observed in other types of patients [39].

#### Microvasculitis

Ischaemic vascular injury caused by microvasculitis is the key factor involved in DRPN. Therefore, DRPN is usually acute in onset and tends to occur unilaterally. Kelkar et al. [41] found that inflammation occurs through immune-mediated processes and proposed that multinucleated microvasculitis occurs due to immune complexes and complement deposition; during this process, patients exhibit multinucleated leukocytes in the epineurium vessels, vascular wall thickening, nonfibrinoid necrosis, and a large number of monocytes or swollen cytoplasmic monocytes. The nuclei of neutrophils change. Vascular permeability increases. Massie et al. [42] also showed that the upper limb nerves of DCRPN patients exhibited more inflammatory lesions than the lower limb nerves of DLRPN patients, and ischaemic injury caused by microvasculitis was the pathophysiological basis underlying DCRPN.

#### Damage to the blood–nerve barrier and blood–dorsal root ganglia barrier

The spinal nerve roots are peripheral nerves consisting of anterior and posterior roots that meet at the intervertebral foramen. The anterior root is a motor branch, and the posterior root is a sensory branch. The axons of the nerve myelin sheath are protected by the nerve bundle membrane around the bundle surrounding the peripheral nerve and dorsal root ganglion, and the epineurium provides a layer of protection outside the nerve bundle membrane. The nerve bundle membrane and the intraneural vessels are the main components of the blood–nerve barrier [43–45]. The endoneurial fluid within the nerve bundles maintains the nerve microenvironment and promotes homeostasis, and this regulatory effect reduces the spread of toxins from the blood to the nerve; this spread is confined to the intraneural vessels and endothelial cells [46–52]. The blood–dorsal root ganglia barrier contains an abundance of sensory nerve cells that are uniquely permeable to allow for greater levels of exchange between nerves and blood. The blood–dorsal root ganglia barrier may respond earlier and be more sensitive to monitoring changes in the chemical components of the internal environment of specific regions of the body. Beamish et al. [53] analysed the rupture of the affected nerve contained within the nerves in diabetic patients and found that the nerve bundles showed greater curvature, reduced numbers of connections, disorganisation at the junctions, and increased numbers of free ends. Based on the anatomical and pathological factors of peripheral nerves, we believe that somatosensory evoked potentials (SEPs) can more rapidly and sensitively reflect abnormal nerve root function during preoperative or intraoperative neurophysiological monitoring in patients with diabetes.

### Impact of diabetes on central nervous system

Diabetes affects not only the peripheral nervous system, but also the central nervous system [15]^.^ The absence of sensory evoked potentials in the brain and spinal cord was found in patients and rodent models with diabetic neuropathies [54–56]. Selvarajah et al. [57] and Eaton et al. [58] also found MRI evidence of spinal cord atrophy. However, the level of evidence in this field is still low. Understanding of the impact of diabetes on the central nervous system provides a new understanding of the diagnosis and treatment of diabetic patients with spinal cervical spondylosis. This interaction may explain the long time required for the recovery of neurological function in patients with cervical spondylotic myelopathy combined with diabetes.

## Anatomical alterations caused by diabetes

Hypertrophic spondylosis is a columnar bone disease characterised by osteophyte formation, mostly at the anterior and lateral aspects of the vertebral body and the intervertebral spaces. As early as 1965, Hájková et al. [59] suggested that the incidence of hyperostotic spondylosis was higher in diabetic patients and that it increased with the age of the patient and the duration of diabetes. In a study of a diabetic gerbil model, Moskowitz et al. [60, 61] found that hyperostosis of vertebral bodies and degeneration of intervertebral discs occur more frequently in the thoracic and lumbar spine, but it can also be observed in the cervical spine. Because of this complication of diabetes mellitus, patients with cervical spondylosis combined with diabetes mellitus are also susceptible to the development of osteophytes in the cervical spine. If such osteophytes form laterally on the vertebral body, they may occupy the space of the intervertebral foramina and lead to mechanical compression of the nerve roots and the blood vessels of the nerve roots. However, it is currently impossible to distinguish the vertebral osteophytes caused by diabetes from those caused by degenerative factors in an imaging examination. Therefore, it remains unclear whether hypertrophic spondylosis is the reason the nerve roots of diabetes patients with cervical spondylosis combined are more prone to proximal compression symptoms.

## The “double crush” syndrome

In 1973, Upton and McComas [62] first proposed the “double crush hypothesis” of a disorder of glucose metabolism that is most common in the upper limb. According to the theory, the peripheral nerves of the upper limbs in diabetic patients are susceptible to entrapment at anatomically narrow sites. Mild entrapment may result in a reduction in axoplasmic flow, but no denervation will occur. If the neuronal conduction of axoplasmic flow is impaired by entrapment at more than two places proximal and distal, clinical symptoms will occur, most commonly at the cervical spine and wrist and cervical spine and elbow. Treatment should primarily address all areas of entrapment that are involved in the nerve conduction process. Metabolic disturbances and microangiopathy due to chronic hyperglycaemia lead to swelling of peripheral nerves [63] and bring about the early fibrinolysis of distal nerves [64]. Due to abnormal glucose metabolism, even in the early stages of diabetes, peripheral nerves may exhibit functional impairment and structural changes. Stamboulis et al. [65] found that patients with diabetes are more prone to local limb neuropathy caused by acute external entrapment. Entrapment neuropathies (EN) are focal lesions that can be common at any stage of diabetes [63, 66] and are the earliest neurophysiological abnormality that occurs. It is especially common in the upper extremities and appears alone even in the absence of systemic polyneuropathy, increasing in frequency with the duration of the disease and the presence of systemic neuropathy. Although there is electrophysiological evidence of focal abnormalities in one or more nerves, these changes may not be accompanied by symptoms of focal neuropathy. For example, the motor and sensory latencies of the median nerve are prolonged, but this finding is similar to the changes seen in carpal tunnel syndrome, making it impossible to distinguish between different types of overlapping entrapment [67].

## Comprehensive preoperative assessment for diabetes patients with cervical spondylosis

For patients with cervical vertebra complicated with diabetes, preoperative clinical diagnosis should be performed very carefully. The occurrence of diabetic neuropathies or the “double crush” phenomenon should be evaluated with especially high caution. Many patients are often considered to exhibit neurological deficits due to entrapment in the cervical region alone. The symmetrical loss of sensation in the distal extremities of both lower limbs, reduction in or absence of physiological reflexes and partial loss of distal muscle strength associated with DPN can challenge the diagnosis of spinal cervical spondylosis. Additionally, the reduced sensory and motor function of the unilateral upper limb associated with DCRPN can challenge the diagnosis of neurogenic cervical spondylosis. Therefore, detailed preoperative physical and electrophysiological examinations are necessary. Electrophysiological diagnosis can be used to distinguish between systemic and focal neuropathy, as well as to demonstrate whether axonal degeneration or changes in demyelination are present and to detect superimposed radiculopathy. The frequency and severity of electrophysiological testing is higher in patients with symptomatic neuropathy, and in patients with asymptomatic diabetics, the mean motor conduction values are 10–30% lower than normal [68]. Cracco et al. [69] also showed that the amplitude of asymptomatic diabetes patients decreased significantly and was greater than the conduction velocity. Dyck et al. [70] showed that the change in demyelination was secondary to the occurrence of axonal degeneration. Neuroultrasound can be used to detect an increase in peripheral nerve cross-sectional area. Regarding laboratory tests, patients with DRPN often exhibit increased levels of rheumatic and serological markers suggestive of nonspecific inflammation, while increased levels of CSF proteins in the cerebrospinal fluid indicate a pathological process extending to the level of the nerve roots [42]_._ New studies have shown that even in the impaired glucose tolerance (IGT) phase, local neuropathy may occur, especially resulting in pain caused by small fibre lesions [71, 72]. The neuropathy associated with IGT is an early form of DPN, and although there is no clear evidence, it is widely accepted that the neuropathy exhibits the same characteristics as DPN in this respect.

## Treatment for diabetes patients with cervical spondylosis

Regardless of the type of diabetic sensorimotor polyneuropathy (DPN or DCRPN), decompression surgery can be used to both reduce pain levels and restore protective sensation, significantly preventing further complications [73]_._ Dellon [74] found that 80% of patients reported significant improvement in pain symptoms after surgical decompression of the nerve roots. In a study by Mondelli et al. [75], 99% of DN patients with carpal tunnel syndrome exhibited an elimination of pain by the surgery. However, whether this finding applies to patients with cervical spondylosis complicated with diabetic neuropathy remains uncertain.

Based on these data, we propose that surgery is necessary for diabetes patients with cervical spondylosis who exhibit a clear diagnosis of indications for surgery. If the patient exhibits double entrapment, bilateral decompression should be performed, rather than just surgery of the cervical spine. However, it is unclear whether all cervical spine surgeries can eliminate symptoms in patients with diabetes combined with cervical spondylosis. Because of the presence of neuropathy, cervical spine surgery may not be able to completely combat the damage to the nerve itself, and new neurological dysfunction may occasionally occur. In patients with cervical spondylosis combined with mild-to-moderate diabetic neuropathy, surgical treatment should be beneficial. However, the effectiveness of surgical treatment in patients with cervical spondylosis combined with severe diabetic neuropathy needs further prospective experimental evidence. However, the possibility of poor surgical outcome and the development of new postoperative neurological dysfunction does exist. In the study of Massie et al. [42], a patient with type 2 diabetes was found to exhibit sensory and motor disorders in the right upper limb and lower limb caused by nerve palsy after general anaesthesia.

For the pharmacological treatment of patients with DPN-induced nerve pain, the main drugs used are tricyclic antidepressants, carbamazepine, gabapentin, pregabalin, tramadol, morphine and oxcarbazepine [8, 76]. Occasionally, electrical spinal cord stimulation may be considered when medication dose not work. Alpha-lipoic acid (ALA) is an effective drug that can be used for a long time [77]. For drug treatment of DCRPN, most of the literature supports the use of early immunotherapy. Krendel et al. [78] showed improvement in patients treated with hormone drugs alone or combined with immunoglobulin or cyclophosphamide drugs. Pascoe et al. [79] found that the immunotherapy group exhibited a greater degree of improvement and a faster speed of recovery than those observed in the nontreatment group. However, the study suggested that glucocorticoids were associated with worsening glycaemic control. Laughlin and Dyck [80] found no serious complications associated with intravenous steroid administration. Opioids are often recommended for the acute pain resulting from DCRPN.

## Conclusions

Diabetes may adversely affect the cervical spinal cord, cervical nerve roots, and peripheral nerves through microangiopathy and microvasculitis. Diabetic neuropathy may occur even in the early stages of diabetes. It is necessary for the surgeon to perform a full evaluation before cervical spine surgery and select a reasonable treatment method; otherwise, poor prognosis of neurological function or the development of new neurological dysfunction may occur. For the treatment of patients with mild and moderate neuropathy with an indication for surgery, surgery remains the treatment of choice. For the treatment of patients with severe neuropathy, drug therapy or surgery combined with drug therapy is preferred to reduce postoperative neurological complications.

## Data Availability

Not applicable.

## Abbreviations

DN: Diabetic neuropathy
DPN: Diabetic polyneuropathy
DRPN: Diabetic radiculoplexus neuropathy
NF-κB: Nuclear factor kappa B
AP-1: Activator protein 1
MAPK: Mitogen-activated protein kinase
DLRPN: Diabetic lumbosacral radiculoplexus neuropathy
DCRPN: Diabetic cervical radiculoplexus neuropathy
DTRN: Diabetic thoracic radiculoneuropathy
CSF: Cerebrospinal fluid
SEPs: Somatosensory evoked potentials
MRI: Magnetic resonance imaging
EN: Entrapment neuropathies
ALA: Alpha-lipoic acid
